# Pleiotropic Effects of mTOR and Autophagy During Development and Aging

**DOI:** 10.3389/fcell.2019.00192

**Published:** 2019-09-11

**Authors:** Kathrin Schmeisser, J. Alex Parker

**Affiliations:** ^1^Max Planck Institute of Molecular Cell Biology and Genetics, Dresden, Germany; ^2^Département de Neurosciences, Université de Montréal, Montreal, QC, Canada

**Keywords:** autophagy, genetics, *C. elegans*, aging, pleiotropy

## Abstract

Autophagy as a ubiquitous catabolic process causes degradation of cytoplasmic components and is generally considered to have beneficial effects on health and lifespan. In contrast, inefficient autophagy has been linked with detrimental effects on the organism and various diseases, such as Parkinson’s disease. Previous research, however, showed that this paradigm is far from being black and white. For instance, it has been reported that increased levels of autophagy during development can be harmful, but become advantageous in the aging cell or organism, causing enhanced healthspan and even longevity. The antagonistic pleiotropy hypothesis postulates that genes, which control various traits in an organism, can be fitness-promoting in early life, but subsequently trigger aging processes later. Autophagy is controlled by the mechanistic target of rapamycin (mTOR), a key player of nutrient sensing and signaling and classic example of a pleiotropic gene. mTOR acts upstream of transcription factors such as FOXO, NRF, and TFEB, controlling protein synthesis, degradation, and cellular growth, thereby regulating fertility as well as aging. Here, we review recent findings about the pleiotropic role of autophagy during development and aging, examine the upstream factors, and contemplate specific mechanisms leading to disease, especially neurodegeneration.

## Introduction

### The Concept of Antagonistic Pleiotropy

The hypothesis of antagonistic pleiotropy was postulated in 1957 by the American evolutionary biologist George C. Williams (1957). It describes the phenomenon when a gene is responsible for more than one phenotypic trait in an organism, whereas at least one trait is beneficial, and one trait is detrimental for this organism. In regard to aging, this often means that a genetic program controlling traits, which is beneficial for development and/or reproduction, is causative for senescence later in life. Thus, antagonistic pleiotropy is often referred to as *Trade-off theory of aging*. A potentially deleterious allele for late life does still underly positive evolutionary pressure in early life, when its effects are beneficial for reproduction of the organism. Therefore, such mutations are not selected against and can accumulate in a population, causing an age-specific decline in organismal performance (Williams, 1957; Moorad and Hall, 2009; Wachter et al., 2013). The antagonistic pleiotropy hypothesis further suggests that aging is a by-product of an investment in development and reproduction, and that genetic variants favored in the fertile stages could cause senescence later in life. Attempts to test the hypothesis of antagonistic pleiotropy has sometimes resulted in contradictory results, and it may be best to consider that the different theories of aging complement one another in terms of describing mechanisms of aging. Since aging is difficult to study in humans, given our life expectancy, ethical concerns and feasibility, data mostly derive from non-human model organisms such as *Drosophila melanogaster* and *Caenorhabditis elegans* (Flatt, 2009, 2011; Anderson et al., 2011). However, developments in DNA sequencing technology within the last decade have led to human genome-wide association studies (GWAS) and allowing detailed insights not only into the genetic basis of disease, but also how they are involved in the aging process. Rodriguez et al. (2017) described the first systematic evidence of senescence genes being associated with pleiotropies in 2017, suggesting a fundamental role of pleiotropy in human aging patterns. They identified 26 early–late onset antagonistic pleiotropies in 19 loci and evidence for positive selection in some of them. For instance, the single-nucleotide polymorphism rs2157719 in the CDKN2A gene is protective for glioma in early life, while exhibiting deleterious effects at older ages, including an increased risk of type 2 diabetes, coronary heart disease, glaucoma and nasopharyngeal cancer. The authors suggest that protection from glioma, a frequent early onset and fatal cancer, has been favored at the costs of increased risk of the deleterious later-onset conditions. Another recent study analyzing GWAS data confirmed that pleiotropy has a very common, if not ubiquitous occurrence in human disease (Chesmore et al., 2018).

### The Mechanistic Target of Rapamycin (mTOR) Pathway and How It Fits in the Theory of Antagonistic Pleiotropy

In the mechanistic target of rapamycin (mTOR) pathway the concept of antagonistic pleiotropy becomes particularly clear (Blagosklonny, 2014). mTOR is needed for development and reproduction, as its functional absence is lethal in embryogenesis (Gangloff et al., 2004; Shiota et al., 2006). Later in life, however, active mTOR drives senescence and increases risk of diseases (Kapahi et al., 2010).

mTOR is an evolutionary highly conserved serine/threonine protein kinase of the PI3K-related kinase family. It forms the catalytic subunit of two distinct protein complexes; mTOR Complex 1 (mTORC1) and mTOR Complex 2 (mTORC2) (Saxton and Sabatini, 2017). mTORC1 consists of three major components – mTOR, Raptor (regulatory protein associated with mTOR), and mLST8 (mammalian lethal with Sec13 protein 8, also known as GßL) (Hara et al., 2002; Kim et al., 2002, 2003). Raptor binds to the TOR signaling motif on several mTORC1 substrates (such as S6 kinase, 4E-BP1, and NPRL2, as described later), therefore facilitating substrate recruitment to mTORC1 (Nojima et al., 2003; Kwak et al., 2016). mLST8, however, is associated with the catalytic domain of mTORC1 and may stabilize the kinase activation loop (Yang et al., 2013). Additionally to the three core components, mTORC1 further contains the two inhibitory subunits PRAS40 (proline-rich Akt substrate of 40 kDa) and DEPTOR (DEP domain containing mTOR interacting protein) (Vander Haar et al., 2007; Peterson et al., 2009). The naturally occurring drug and mTOR name giver rapamycin directly inhibits mTORC1, whereas mTORC2 is insensitive to acute rapamycin treatment. Similar to mTORC1, mTORC2 also comprises of mTOR and mLST8, but instead of Raptor it contains Rictor (rapamycin insensitive companion of mTOR), an unrelated protein that potentially serves an analogous function (Sarbassov et al., 2004). Furthermore, mTORC2 contains DEPTOR and the regulatory subunits mSin1 and Protor1/2 (Yang et al., 2006; Pearce et al., 2007; Peterson et al., 2009). mTORC1 and 2 regulate different cellular processes in response to environmental clues, however, mTORC1 controls the balance between anabolism and catabolism, most notably in this context autophagy, and will therefore be the focus of this review.

The mTOR pathway is activated by hormones, growth factors, and nutrients such as glucose, amino acids, and fatty acids. These stimuli, except for amino acids, activate mTORC1 via the tuberous sclerosis TSC1–TSC2 complex and the small GTPase Ras homolog enriched in the brain (Rheb). Hormones such as insulin and growth factors including insulin-like growth factors (IGFs) signal to mTORC1 through the insulin receptor/phosphoinositide 3-kinase/AKT signaling pathway, where mTORC1 is activated by AKT/protein kinase B (Sarbassov et al., 2006). Amino acids promote mTORC1 translocation to the lysosomal membrane, where it becomes activated upon conversion of RagA or RagB GTPases from a GDP- to GTP-bound state with the help of the folliculin tumor suppressor (Bar-Peled et al., 2012; Tsun et al., 2013).

Subsequently, the mTOR pathway regulates transcription factors such as FOXO, FOXA, NRF, NF-κB, SREBPs, and TFEB, and induces ribosome biogenesis, protein synthesis, cellular growth and secretion of pro-inflammatory and mitogenic factors, and inhibits autophagy when food is plentiful (Wullschleger et al., 2006; Peterson et al., 2011; Zhou et al., 2018). In general, under unfavorable conditions, mTOR is inhibited, leading to the inhibition of global protein synthesis and major savings of energy.

There are two major downstream targets of mTORC1; the eukaryotic initiation factor 4E-binding protein (4E-BP) and the ribosomal subunit p70S6 kinase 1 (S6K). Both initiate mRNA translation initiation and thereby protein synthesis (Hay and Sonenberg, 2004; Sonenberg and Hinnebusch, 2009). mTORC1 directly phosphorylates S6K, leading to subsequent phosphorylation and activation by PDK1. S6K then phosphorylates and activates several substrates that promote mRNA translation initiation, including a positive regulator of the 5′cap binding eIF4F complex called eIF4B (Holz et al., 2005). S6K also promotes the degradation of PDCD4, an inhibitor of eIF4B, via phosphorylation, and increases translation efficiency of spliced mRNAs (Dorrello et al., 2006; Max et al., 2008). 4E-BP is unrelated to S6K and inhibits translation by binding and sequestering eIF4E to prevent formation of the eIF4F complex. mTORC1 phosphorylates 4E-BP to trigger its dissociation from eIF4E, enabling 5′cap-dependent mRNA translation (Gingras et al., 1999).

mTOR furthermore enables cellular growth by providing sufficient lipids for membrane formation and expansion. *De novo* lipid synthesis is promoted by mTORC1 through activation of the sterol responsive element binding protein (SREBP) transcription factors, which subsequently regulate expression of genes involved in cholesterol and fatty acid synthesis (Porstmann et al., 2008; Duvel et al., 2010). Furthermore, mTORC1 increases the translation of the transcription factor HIF1α, thus facilitating cell growth by inducing a shift in glucose metabolism from oxidative phosphorylation to glycolysis, which has been shown to incorporate nutrients into new biomass (Duvel et al., 2010). Recent studies have found that mTORC1 also promotes nucleotide biogenesis required for DNA replication and ribosome synthesis in growing and proliferating cells by facilitating purine synthesis (Ben-Sahra et al., 2016).

### mTOR and Autophagy

As outlined above, mTOR promotes anabolic cellular processes leading to growth. This is further facilitated by the suppression of protein catabolism, most notably autophagy. Autophagy is a basic catabolic process in the cell that degrades damaged organelles or dysfunctional proteins to gain energy or free amino acids. Three different types of autophagy have been defined: microautophagy, chaperon-mediated autophagy and macroautophagy, with the latter being the predominant form (referred to as autophagy hereafter). During the first step of autophagy, the cytoplasmic components that are to be degraded are engulfed in a double membrane, building the so-called autophagosome. Autophagosomes then fuse with lysosomes (or vacuoles in plant and yeast cells), exposing their contents to hydrolases, which catalyze degradation (Levine and Klionsky, 2004; Mizushima and Komatsu, 2011). Originally believed to be stress-induced, it is now clear that a cell needs basal levels of autophagy to maintain homeostasis, however, the process is strongly activated upon stress. Particularly the absence of nutrients and growth factors as in calorie restriction are strong inducers of autophagy. Other forms of stress that induce autophagy are DNA or protein damage, reactive oxygen species (ROS), or pathogens. In eukaryotes, autophagy is a tightly regulated process with mTOR being one of the most important regulators (Neufeld, 2010). Upon stress, mTOR is inhibited, leading to the induction of autophagy in yeast, *C. elegans*, *Drosophila*, and mammals (Noda and Ohsumi, 1998; Ravikumar et al., 2004; Hansen et al., 2008; Kenyon, 2010).

On a molecular level, autophagy induction is mediated by activation of ULK1 (Atg1 in yeast, UNC-51 in *C. elegans*), a serine/threonine kinase that forms a complex with ATG13, FIP2000, and ATG101 (Chang and Neufeld, 2009; Nazio et al., 2013). When nutrients are plentiful, mTOR-dependent phosphorylation of ATG13 suppresses the ULK1 complex, thereby preventing its activation by AMPK, a key activator of autophagy (Kim et al., 2011). Hence, the relative activity of AMPK and mTOR, which can be seen as counterplayers in the cell, determine autophagy induction and activity. Under nutrient deprivation or stress, mTORC1 is inhibited by various pathways, increasing ULK1 activity, leading to autophagosome nucleation and elongation, early activation steps in the autophagic process (Rabinowitz and White, 2010). mTOR furthermore regulates autophagy by phosphorylating and thus inhibiting the nuclear translocation of the transcription factor TFEB, which shifts gene expression toward lysosomal biogenesis and the autophagy machinery (Martina et al., 2012; Roczniak-Ferguson et al., 2012; Settembre et al., 2012). Additionally, AMPK has been found to promote autophagy through mTOR-dependent TFEB activation and increasing the levels of the arginine methyltransferase (CARM1), an important cofactor for TFEB transcription (Shin et al., 2016; Young et al., 2016). In the *Drosophila* larval fat body, a functional homolog of vertebrate liver and adipose tissue, starvation induces autophagy via inactivation of mTOR and its upstream regulators phosphoinositide 3-kinase and Rheb (Scott et al., 2004). Strikingly, the same study has found that S6K activity is required to induce autophagy, contradicting the predominant opinion that S6K acts solely as autophagy suppressor. In line with these findings, rapamycin-induced inhibition of mTOR enhances the kinase activity of ULK1, whereas mTOR activation through Rheb overexpression represses ULK1 (Jung et al., 2009). Moreover, it has been suggested that mTOR indirectly inhibits autophagy through the phosphorylation of autophagy/Beclin-1 regulator 1 (AMBRA1), which prevents ubiquitination of ULK1, causing ULK1 self-association, stabilization, and enhancement of its kinase activity under starvation (Nazio et al., 2013). A recent study has found that inflammation processes induced by lipopolysaccharides activate mTOR and inhibit autophagy via the upstream toll-like receptor 4 (TLR4) signaling pathway and downstream NF-κB activation (Zhou et al., 2018). In addition to these findings, several other mechanisms have been proposed as to how mTOR impacts autophagy. These include mTOR-dependent regulation of death-associated protein 1 (DAP1), a suppressor of autophagy, and WIPI2, a mammalian ortholog of Atg18 (a regulator of autophagosome formation in yeast), which was identified as potential mTOR effector (Koren et al., 2010; Hsu et al., 2011).

Underlying this classic example of a pleiotropic pathway, mTOR-mediated autophagy regulation is prone to be a pleiotropic process itself, and recent research strongly suggests that activity of autophagy has various effects ranging from beneficial to detrimental depending on the state of development and aging.

### mTOR-Independent Regulation of Autophagy

Apart from the regulation of autophagy by mTORC1, various mTOR-independent autophagy pathways have been described. A major pathway in this regard is the inositol signaling pathway, as elevation of intracellular inositol or Ins(1,4,5)P3 levels can inhibit autophagosome formation (Sarkar et al., 2005). Ins(1,4,5)P3 acts as a second messenger and binds to its receptors (IP3R) on the endoplasmatic reticulum, thereby releasing Ca^2+^ into the cytoplasm that elicits a range of cellular responses, including regulation of autophagy (Criollo et al., 2007). It has been shown that elevation in intracellular Ca^2+^ has complex effects in impairing autophagy, which affects both autophagosome formation and autophagosome–lysosome fusion (Williams et al., 2008; Ganley et al., 2011). A screen of FDA-approved drugs also revealed several compounds that regulate autophagy in an mTOR-independent manner via the modulation of cytosolic Ca^2+^ (Zhang et al., 2007). Furthermore, several studies have shown that elevation of intracellular levels of the second messenger cAMP inhibits autophagy (Noda and Ohsumi, 1998), as well as the JNK1/Beclin-1/PI3KC3 pathway (Pattingre et al., 2005), within which the leucine rich repeat kinase 2 (LRRK2) plays a major role in controlling autophagy (Manzoni et al., 2016). Additionally, several small molecules have been identified that regulate autophagy mTOR-independently, many of them with an unknown mechanism of action. For instance, trehalose, a disaccharide found in various non-mammalian species, is a potent autophagy activator (Sarkar et al., 2007). Furthermore, ROS have been reported as early inducers of autophagy upon nutrient deprivation and other circumstances, where the p62/Keap1/Nrf2 pathway is of major importance (Ristow and Schmeisser, 2014; Filomeni et al., 2015). Notably, ROS can regulate autophagy both mTOR-dependently and independently.

## Age-Related Changes in Autophagy

**FIGURE 1:**
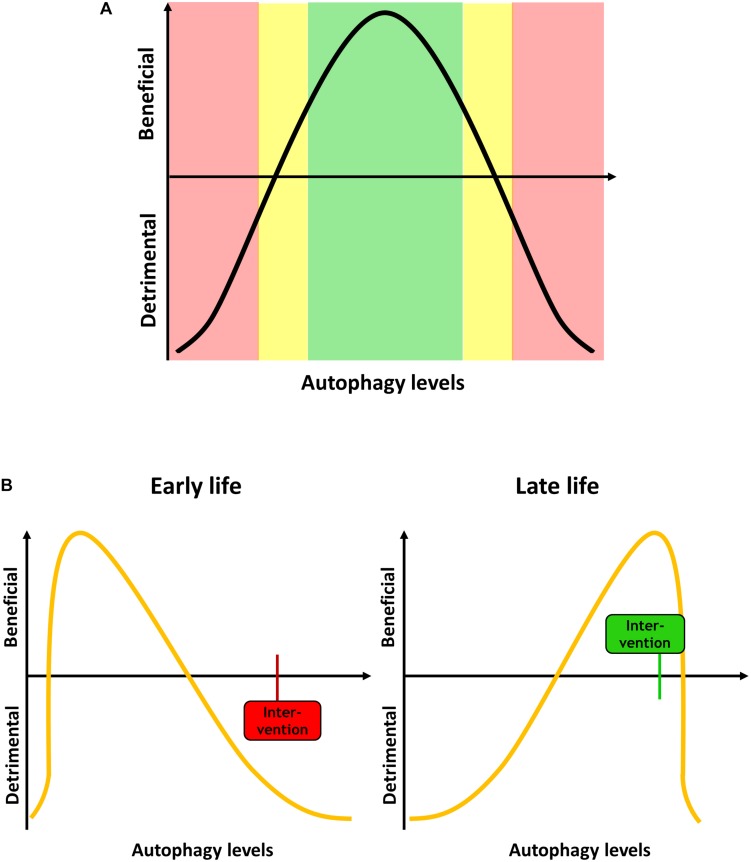
**(A)** Autophagy levels have to be tightly regulated, and both too high or too low levels can be detrimental for the cell and the organism. **(B)** Autophagy demand changes during aging. During development and early life, when less dysfunctional proteins and organelles occur in the cell, low levels of autophagy are beneficial. An intervention increases the autophagy, such as mTOR inhibition, can be detrimental. In late life, high autophagy levels are beneficial, and an intervention that in early life would be deleterious can become beneficial.

It is now generally accepted that autophagic regulation and maintenance underlies major changes during lifespan, a phenomenon that has been studied mainly in model organisms such as yeast, *C. elegans* and *Drosophila*, but also rodent models and aged mammalian tissue. However, it is not clear whether increases or decreases in autophagy are causally related to aging and age-associated impairment of cellular function and organismal health (Levine and Kroemer, 2008). There is considerable evidence that the efficiency of autophagic degradation declines with age, which could lead to an accumulation of dysfunctional organelles and damaged proteins that contribute to cellular aging (Cuervo, 2008; Ghosh et al., 2016; Ott et al., 2016; Zhang et al., 2017; Zhou et al., 2017; Li et al., 2018). In contrast, it has been suggested that activation of autophagy during aging leads to enhanced clearance of aged cellular components, which improves health span (Escobar et al., 2019; Miyamoto, 2019; Shi et al., 2019; Singh et al., 2019). In general, it is suggested that autophagic processes are increased in animals with a long lifespan, and that autophagy is essential to mediate said lifespan extension (Chang et al., 2017; Arensman and Eng, 2018). Vice versa, this suggests that impaired autophagy activity causes rapid aging and age-related diseases, which indeed may be the case for diseases like diabetes, cardiovascular disease, neurodegenerative diseases and cancer (reviewed in Saha et al., 2018). Furthermore, if autophagy is linked to antagonistic pleiotropy, the beneficial effect of increased autophagy in later life would insinuate that high autophagic activity is problematic during development, or that low autophagy is beneficial during development and detrimental in aging (Figure 1).

Methodological advancements in genetics and imaging technology now allow for the study of autophagy in great detail. A means of quantifying autophagy in different cell types is monitoring autophagosome formation by expressing fluorescently marked Atg8 (in yeast)/LGG-1 (in *C. elegans*), which are orthologs of mammalian LC3 (Klionsky et al., 2016). When not being employed in autophagy, Atg8/LGG-1 is distributed evenly in the cytosol. When autophagosomes form, Atg8/LGG-1 is cleaved, conjugated to phosphatidylethanolamine and inserted into the vesicle double membrane. Autophagosomes then occur as puncta and can be quantified. However, as this only reports the steady-state and not the rate of autophagosome formation and metabolization into autolysosomes, important information could be lost. Therefore, a tandem-tagged mCherry-GFP-Atg8/LGG-1 reporter has been developed that monitors both autophagosomes (yellow [green/red] puncta) and autolysosomes (red puncta as GFP fluorescence quenches in the acidic autolysosome environment), the so-called autophagic flux (Kimura et al., 2007; Manil-Segalen et al., 2014; Mauvezin et al., 2014; Klionsky et al., 2016). Several studies have addressed the autophagic flux at different stages during *C. elegans* lifespan. It was suggested that autophagic functionality increases during development until day 2 of adulthood (Chapin et al., 2015). Another study found that autophagosome formation increases up to day 10 of adulthood in different tissues, including neurons, intestine, muscle, and pharynx (Chang et al., 2017). However, it was observed that the flux became increasingly dysfunctional with age and the observed increase in autophagic vesicles was due to impaired degradation and accumulation. This was confirmed by other researchers, who identified a blockage of the late autophagic flux, leading to accumulation of autophagosomes (Wilhelm et al., 2017). In a study using *C. elegans* in our laboratory we found a similar phenomenon: Autophagosome numbers increased up to day 9 of adulthood, and this cannot be regulated by starvation, suggesting that autophagy is “out of control” as the animals get older (Schmeisser and Parker, 2018).

To study the effect of aging on autophagy in humans, several human tissues have been examined. Studies in human skin fibroblasts found that the number of autophagosomes and amount of LC3 is not significantly different between young and old cells (Demirovic et al., 2015; Kim et al., 2018), but that autophagic degradation is impaired (Tashiro et al., 2014). Furthermore, human skeletal muscle has been shown to be robust to changes in autophagy markers during aging, in contrast to several studies in mice and rats where a divergent regulation of autophagy with aging was reported (Garcia-Prat et al., 2016; Fan et al., 2017; Zhou et al., 2017; Dethlefsen et al., 2018). For instance, autophagosome accumulation and dysfunctional degradation have been reported repeatedly in aging muscle; a phenomenon that had also been observed in post-mortem brain samples from old tauopathies patients (Piras et al., 2016). Other studies in brain tissue from mammalian models and humans have found conflicting results and further research is needed to shed light on this topic (reviewed in Loeffler, 2019). Autophagy has also been shown to play a major role in physiology, development, and aging of the eye, and many eye diseases that mostly occur with aging display alterations in autophagic pathways (reviewed in Boya et al., 2016). In cardiac tissue, autophagy dysfunction has been reported in aging rodent models and in a limited number of human studies (reviewed in Linton et al., 2015).

Some of the effects of inefficient or dysfunctional autophagy during aging might be mediated by rubicon (Run domain Beclin-1 interacting and cysteine-rich containing protein), which is a highly conserved negative regulator of autophagy. Rubicon expression is upregulated during aging in worms, flies, and mice, leading to decreased autophagy via inhibiting autophagosome-lysosome fusion and endocytic trafficking (Matsunaga et al., 2009; Nakamura et al., 2019).

## mTor-Dependent Autophagy in Development and Aging − a Bad Start Compensates for Later

A classic inhibitor of mTOR is rapamycin (Sirolimus), which is used as immunosuppressor and antiproliferative drug in human medicine. Inhibition of mTOR by rapamycin and other interventions, which potently induces autophagy, has been shown to improve healthy aging and lifespan throughout various species, however, at the cost of development. Vice versa, active mTOR is beneficial in development at the cost of aging. Classic antagonistic pleiotropy of mTOR inhibition was first described in *C. elegans* (Vellai et al., 2003): Mutants of LET-363 (LET for lethal), the nematode ortholog of mTOR, arrest before they become fertile as L3 larvae but have a strikingly extended lifespan. A similar pleiotropic phenotype had been described in mutants of the insulin/IGF-1 receptor DAF-2: while their fertility is reduced, they show remarkable longevity (Tissenbaum and Ruvkun, 1998). Interestingly, the lifespan of DAF-2 animals cannot be further extended when mTOR is inhibited, suggesting a common mechanistic pathway (Vellai et al., 2003). A direct interaction between insulin and mTOR signaling was shown by Jia et al. (2004). Later studies found that autophagy is essential for both the longevity caused by DAF-2 mutation and mTOR inhibition (Melendez et al., 2003; Hansen et al., 2008; Toth et al., 2008). In other species, mTOR inhibition resembles the pleiotropic phenotypes observed in *C. elegans*, and it is now clear that autophagy is a major mediator of longevity due to mTOR inhibition in yeast (Alvers et al., 2009; Matecic et al., 2010), *Drosophila* (Kapahi et al., 2004; Bjedov et al., 2010) and mice (Harrison et al., 2009). On the other hand, however, this longevity again comes at the price of early developmental caveats: In budding yeast, the deletion of six genes implicated in the TOR signaling pathway extended lifespan, however, some deletion mutants were slow growing and deletion of both paralogs could be lethal (Kaeberlein et al., 2005). In *Drosophila*, a homozygous mutation in S6K leads to developmental delay and a reduction in body size (Montagne et al., 1999; Um et al., 2006), while overexpression of the constitutively active form of S6K caused significant shortening of the lifespan with no developmental constraints (Kapahi et al., 2004). Rapamycin treatment in female flies caused potent lifespan extension, but significant reduction of brood size (Bjedov et al., 2010). Furthermore, mTOR has been established in mammals as a central developmental regulator of cell, organ, and organismal size in mammals (reviewed in Saxton and Sabatini, 2017). Conversely, mice with a constitutively active allele of RagA that prevents mTORC1 inhibition by nutrient starvation develop normally, but do not survive starvation periods because they cannot switch from an anabolic to a catabolic state (Efeyan et al., 2013).

A way around the negative effects of antagonistic pleiotropy in mTOR-dependent autophagy was tested in Harrison et al. (2009), when mice were given rapamycin to inhibit mTOR only from the advanced age of 600 days, which in human resembles about 60 years, and they still showed enhanced lifespan with no major side effects. Notably, the lifespan could not further be increased when rapamycin treatment started at a younger age of 270 days (Harrison et al., 2009).

## Pleiotropic Effects of mTor-Dependent Autophagy in Neurodegeneration

**FIGURE 2:**
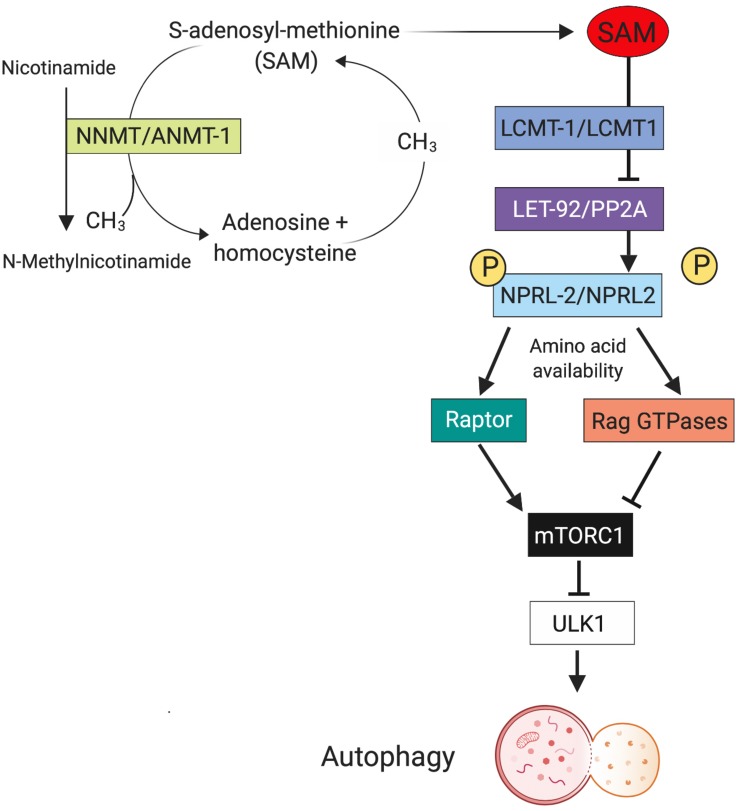
Metabolic regulation of pleotropic autophagy. One mechanism that may contribute to the differential effects of autophagy on aging phenotypes is metabolism of *S*-adenosyl methionine (SAM). The enzyme nicotinamide *N*-methyl-transferase (NNMT) methylates nicotinamide (NAM) to *N*-methylnicotinamide (MNA) SAM as the methyl group donor. This reduced cellular concentration of SAM precludes it from functioning in the LCMT1/PP2A/NPRL2 pathway, that in turn regulates autophagy. Thus, the relative expression levels of enzymes like NNMT during aging can influence autophagy with have profound effects on neuronal function and survival. Created with BioRender.com.

Neurons are particularly vulnerable to dysregulated autophagic processes because they are post-mitotic cells and unable to undergo cytokinesis, so damaged organelles and aggregated proteins are not being diluted by cell divisions (Nixon, 2013). A common hallmark for many neurodegenerative diseases are aggregated and dysfunctional proteins, which would be degraded by normal autophagy. Indeed, impaired autophagy has been reported for many major neurodegenerative diseases such as Alzheimer’s disease (Boland et al., 2008), Parkinson’s disease (Michel et al., 2016), Huntington’s disease (HD) (Martinez-Vicente et al., 2010), and amyotrophic lateral sclerosis (Chen et al., 2012). Whereas neurodegenerative diseases usually occur later in life, impaired autophagy also plays a key role in neurodevelopmental or early onset psychiatric disorders, such as autism spectrum disorder and schizophrenia (Bowling and Klann, 2014; Merenlender-Wagner et al., 2015; Kim et al., 2017). In all the mentioned disorders, it is not clear if increased or decreased autophagy activity is the culprit of disease initiation or progression, and we propose that there might be a role for antagonistic pleiotropy in autophagy underlying neuronal disease. Our previous research has shown that increased neuronal autophagy leads to lifespan extension and lower levels of neuronal cell loss in old *C. elegans* (15 days, which in humans may resemble an age of around 65 years), however, at the cost of reduced fertility and behavioral abnormalities in young animals (Schmeisser and Parker, 2018). The molecular pathway that is responsible for autophagy comprises of a leucine carboxyl methyltransferase (LCMT1), which methylates and thus activates the catalytic subunit of protein phosphatase 2A (PP2A). Methylated PP2A subsequently dephosphorylates the NPR2-like GATOR1 complex subunit that is part of a complex that controls autophagy via the regulation of mTOR (Sutter et al., 2013; Laxman et al., 2014). A recent study in tissue culture has found that dependent on the phosphorylation status of NPRL2 and amino acid availability, NPLR2 binds either to Raptor, which will activate mTORC1 when there are plentiful amino acids and NPRL2 is phosphorylated. When NPRL2 is dephosphorylated and amino acids are scarce, it will bind to RagGTPases, which will inhibit mTORC1 leading to activation of ULK1 and therefore autophagy (Kwak et al., 2016). Furthermore, we and others have found an important role for S-adenosyl-methionine (SAM) in this regard, as SAM is used as methyl group donor in the methylation catalyzed by LCMT1 (Sutter et al., 2013; Schmeisser and Parker, 2018). We speculated that SAM serves as nutrient sensor in the cell, because we found that SAM levels are increased when *C. elegans* are starving and that low SAM will induce autophagy in the cell (Figure 2). This could also be mediated by the recently discovered SAMTOR, a previously uncharacterized protein, which interacts with mTOR and inhibits mTOR signaling when SAM concentration is low (Gu et al., 2017). Furthermore, *C. elegans* with a deletion mutation in S-adenosyl methionine synthetase (*sams-1*), the enzyme responsible for bulk SAM production in the worm, show significantly decreased SAM levels and increased autophagy, which is accompanied by extremely reduced brood size, slow growth, and longevity – classical antagonistic pleiotropy (Hansen et al., 2005; Schmeisser and Parker, 2018).

Another example for this phenomenon in neurodegeneration is found in patients of HD, a rare autosomal dominant disease, with an onset in post-reproductive stages and a strong involvement of autophagy (Son et al., 2012). Several decades ago it was reported that individuals affected by HD have increased reproductive fitness, even before the underlying molecular mechanism of the intergenerationally increasing number of CAG trinucleotide repeats in HD was known (Shokeir, 1975; Albin, 1993; MacDonald et al., 1993). Shokeir reported in 1975 that HD patients have 39% more children than healthy controls (Shokeir, 1975). Furthermore, HD patients have significantly lower rates of some cancers, which was associated with higher expression of the tumor suppressor gene p53. p53 induces higher apoptosis rates, and high apoptosis could also be linked to neurodegenerative episodes in HD (Sorensen et al., 1999; Eskenazi et al., 2007). Strikingly, increasing autophagy via rapamycin or genetic inhibition of mTOR has been shown to be neuroprotective in cell, fly and mouse models of HD as protein aggregates are degraded and polyglutamine expansion toxicity is reduced (Ravikumar et al., 2004; Sarkar et al., 2009; Martin et al., 2015). This could also be achieved by activation of AMPK or small molecules, both of which inhibit mTOR (Tsvetkov et al., 2010; Walter et al., 2016). Interestingly, a recent study found that induction of neuronal autophagy in a mouse model of HD could also be achieved by intermittent fasting, an intervention known to inhibit mTOR (Ehrnhoefer et al., 2018).

## Conclusion

Given the number of remarkable new findings regarding autophagy that are being published continuously (Ezcurra et al., 2018; Saito et al., 2019), the future will show whether the link between mTOR-mediated autophagy and pleiotropic autophagy will be sustained. For now, many studies suggest this may be the case. During reviewing the current literature, we noticed that many researchers studying the processes of autophagy focus on either the developmental side, or on aging. This is understandable, given the depth of research in both topics, but also the technical demands of these fields. However, it makes it difficult to draw a reliable conclusion when antagonistic pleiotropic effects of autophagy are not being described within the same study but have to be pieced together in a rather uncontrolled fashion.

Even in model organisms like *C. elegans* that are relatively easy to use as model for aging, technical difficulties can occur. First, when a worm population starts to die at around day 12 of adulthood, every study of the population after that day involves dead individuals, and every study of individuals is cherry-picking, leading to potential bias in the results. Second, a strong background fluorescence especially in the intestinal tract of *C. elegans* that increases with aging limits the use of many fluorescence markers, and these are the major tools we have available so far to visualize the autophagy process in the cell. The generation and establishment of better tools (and potentially models) could allow to accurately monitor spatiotemporal regulation and functionality of autophagy within different tissues in development and aging, to gain deeper insights into the larger overall hypothesis of antagonistic pleiotropy.

## Author Contributions

KS wrote the manuscript. JP provided direction and edited the manuscript.

## Conflict of Interest Statement

The authors declare that the research was conducted in the absence of any commercial or financial relationships that could be construed as a potential conflict of interest.
